# Effectiveness of Facebook Remote Live-Streaming-Guided Exercise for Improving the Functional Fitness of Community-Dwelling Older Adults

**DOI:** 10.3389/fmed.2021.734812

**Published:** 2021-09-23

**Authors:** Shao-Hsi Chang, Li-Ting Wang, Ting-Yu Chueh, Ming-Chun Hsueh, Tsung-Min Hung, Yu-Wen Wang

**Affiliations:** ^1^Department of Physical Education and Sport Sciences, National Taiwan Normal University, Taipei, Taiwan; ^2^Graduate Institute of Sport Pedagogy, University of Taipei, Taipei, Taiwan; ^3^Institute for Research Excellence in Learning Sciences, National Taiwan Normal University, Taipei, Taiwan; ^4^Office of Physical Education, Chang Gung University, Taoyuan, Taiwan

**Keywords:** elderly, live stream, home-based exercise, fitness, physical activity

## Abstract

**Background:** The aim of this study was to determine the effect of Facebook remote live-streaming-guided exercise on the functional fitness of community-dwelling older adults.

**Method:** This study used a non-randomized controlled design with single-blinding (outcome assessors). Older adults (mean age = 70.36 ± 4.51 years) were assigned to either the experimental group (*n* = 39) or the control group (*n* = 34). The experimental group participated in a 75-min Facebook remote live-streaming-guided exercise routine twice a week for 8 weeks at home, whereas the control group maintained their original lifestyle without any intervention. Functional fitness was assessed using the Senior Fitness Test, which assessed upper and lower limb flexibility and muscle strength, cardiorespiratory fitness, and balance. The test was administered before and after the intervention.

**Results:** The results revealed that an 8-week Facebook remote live-streaming-guided exercise intervention increased lower limb flexibility and muscle strength and cardiorespiratory fitness in community-dwelling older adults.

**Conclusion:** The current findings suggest that a home-based exercise program using the Facebook platform may be a feasible method to broadly improve the functional fitness of community-dwelling older adults.

## Introduction

According to the United Nations, the number of individuals aged 65 years and over is projected to make up 16% of the world's population by 2050 (1). However, this dramatic increase in life expectancy comes with a proportionate decrease in the quality of life of older adults. This decrease in quality of life may be attributed to the decline in functional fitness associated with aging (2, 3). Functional fitness is defined as having the physiological capacity to safely and independently perform normal everyday activities. It includes upper and lower limb flexibility and muscle strength, cardiorespiratory fitness, and balance (4). Previous studies have shown that lower levels of functional fitness are associated with a higher risk of falling (5), chronic disease (6), and cognitive impairment (7), which result in major social and medical challenges to society (8). Therefore, maintaining and increasing functional fitness in older adults are crucial issues.

As regular physical activity is a viable means of maintaining/improving the functional fitness of older adults (9), the second Physical Activity Guidelines for Americans in 2018 recommend that older adults above the age of 65 years engage in at least 150 min per week of moderate-intensity physical activity (PA) (10). However, several factors may affect older adults' intention to participate in exercise, such as the weather or the distance between their residence and the place they exercise (11). These may have unintended negative effects of reducing physical activity and increasing sedentary behavior, which may, consequently, cause deterioration of the functional fitness of older adults (12). Recently, the COVID-19 pandemic has abruptly altered how we carry out our daily lives. Many countries have implemented policies for social distancing and varying degrees of quarantine or isolation at home (13). Such strategies further deteriorates the motivation for engagement in physical activity (14, 15). Although conducting home-based exercises could be an alternative and a feasible method for eliminating barriers to engage in physical activity, one of the challenges of implementing home-based exercise training is the provision of instructions or feedback during the exercise intervention.

Facebook may be a feasible platform for implementing a home-based exercise training program for older adults. Facebook is the most popular social media platform worldwide (16). The number of older adult members of Facebook in Taiwan is ~600,000 (17). Facebook allows the provision of effective instructions during exercise interventions via the live-streaming tool. That is, instructors are able to offer real-time feedback or instructions to participants to increase the effectiveness of the exercise regime for increasing functional fitness. Although some studies (18, 19) have utilized home-based exercise intervention to improve the functional fitness of older adults using telepresence platforms (e.g., Skype), the effect of tele-exercise on functional fitness is inconclusive and limited, as it has only been shown to increase lower limb flexibility or muscle strength. The possible reasons for these inconclusive results may be the small sample sizes used (e.g., exercise group = 11, control group = 12) and the participants' characteristics (healthy elderly men vs. elderly women with a high risk of falling). To the best of our knowledge, there are no home-based exercise interventions designed to use remote live streaming through Facebook to increase the functional fitness of older adults. Previous studies have indicated that 8 weeks of traditional exercise intervention yield positive effects on the functional fitness of older adults (20–22). Therefore, the aim of this study was to investigate the effects of an 8-week Facebook remote live-streaming-guided exercise program on the functional fitness of community-dwelling older adults. We hypothesized that this program would improve the functional fitness of the participants.

## Methods

### Participants

Participants were recruited from several districts of Taipei City through advertisements. Participants were eligible for the study if they (1) were aged above 65 years and were living on their own in the community; (2) had a Facebook account; (3) were capable of walking without an assistance device; (4) were free from any medical condition listed on the Physical Activity Readiness Questionnaire (PAR-Q) (23); and (5) had normal or corrected-to-normal vision. One hundred and ten older adults volunteered to participate in this study. However, 20 of these volunteers were excluded due to (1) an age <65 years (*n* = 6) or (2) the presence of a disease on the PAR-Q list (*n* = 14). This study utilized a single-blinded (outcome assessors) non-randomized controlled design. Fifty participants were assigned to the experimental group and 40 were assigned to the control group based on their willingness and Facebook usage habits. This sample size was satisfactory to attain a power of 0.80 at an alpha level of 0.05 for F tests with moderate effect sizes that were reported in previous studies (24–26). All participants provided written informed consent in the format approved by the Research Ethics Committee of the National Taiwan Normal University. Data were collected between December 2019 and January 2020. All eligible participants were compensated with a gift certificate and a yoga mat after completing the study.

### Intervention

**Figure 1:**
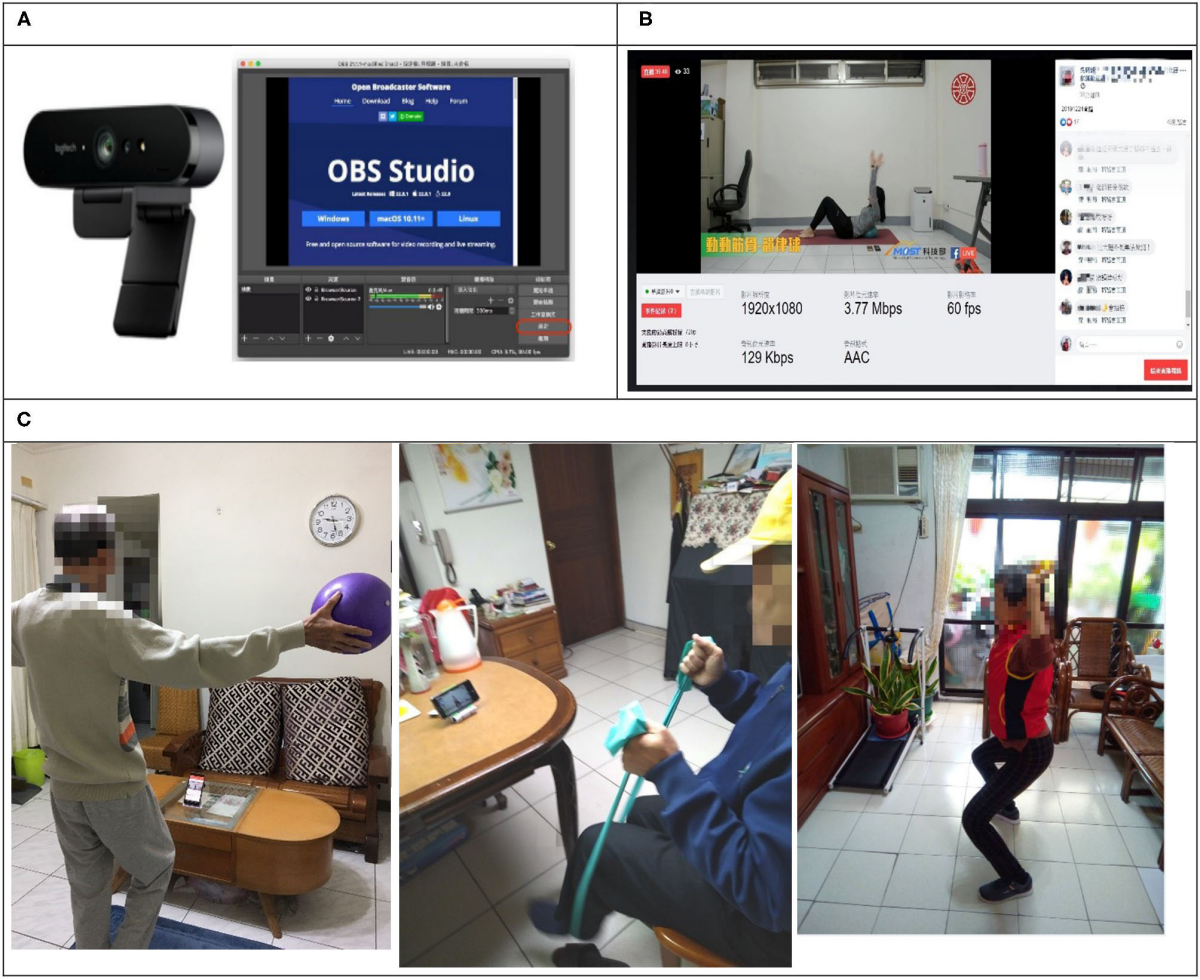
Facebook remote live-streaming-guided exercise. **(A)** Equipment and software for live-streaming exercises. **(B)** Instructor's remote exercise conferencing display. **(C)** Pictures of participants following the instructor's movements.

For the experimental group, the exercise intervention consisted of a twice-weekly training session for 8 weeks. Considering the effects of the intervention on functional fitness, an attendance rate higher than 80% was mandatory to be considered in the final sample of the study. Participants in the experimental group used a mobile phone or computer to access the Facebook platform and participate in the exercise program. To allow an instructor to clearly demonstrate the designed movements and provide instructions to participants, a technician created a Facebook group and utilized a camera (Brio 4k Pro Webcam; Logitech, Lausanne, Switzerland) and remote live-streaming software (Open Broadcaster Software Studio, https://obsproject.com/download). That is, the instructor and participants were all in the same Facebook group during the exercise intervention (Figure 1). All exercise courses were instructed by one of the authors (LTW), who has substantial experience in instructing older adults. The exercise instructions followed those of previous studies (27, 28) that focused on improving the functional fitness of older adults. For flexibility training, participants performed 10 stretching movements on a yoga mat, holding each for 15 s. For muscle strength training, participants performed six movements, using an elastic band, that focused on the biceps and quadriceps muscles, with 8–10 repetitions of each movement. For cardiovascular fitness training, participants were instructed to perform an aerobic dance for 20–30 min. For balance training, participants were instructed to perform calf raises and stand on one foot, with a progressively decreasing amount of support. The total duration of each exercise session was 75–90 min. Participants in the control group received no intervention and were instructed to maintain their normal routine of daily activities.

### Functional Fitness Test

The Senior Fitness Test was used to assess the functional fitness of the participants (29). The effectiveness and reliability of this tool have been widely documented (4). All participants were required to complete functional fitness before and after the intervention at the same community activity center by assessors who were blinded to group allocations. The test has six items and evaluates four physical functional dimensions using the following movements: back scratch, chair sit-and-reach, arm curl, chair stand, 2-min step, and 8-foot starting distance.

- Back scratch. The back scratch test assesses upper body flexibility. Participants were asked to stand and place their hand behind their back. The distance that their hands overlapped behind them was then measured.
- Chair sit-and-reach. This movement evaluates lower body flexibility. To perform this test, participants sat on a chair, keeping one leg straight out, and stretched their hands toward their toes. This pose was held for 2–3 s.
- Arm curl. The arm curl movement assesses upper body strength. Men used an 8-lb dumbbell, and women used a 5-lb dumbbell. The number of biceps curls that could be completed in 30 s was recorded.
- Chair stand. The chair-stand test evaluates lower body muscle strength. Participants were asked to fold their arms across their chest, stand from their chair, and return to the sitting position. This was repeated for 30 s.
- 2-min step. The 2-min step exercise evaluates cardiorespiratory fitness. Participants were asked to raise their knee to the midway point between the patella and the iliac crest as many times as possible within 2 min.
- 8-foot up-and-go. The 8-foot up-and-go exercise evaluates balance and agility. The time taken for a participant to rise from a seated position, stand up, walk a distance of 8 feet (2.44 meters) from the chair, and return to a seated position in the chair was measured. Their fastest time was recorded.

### Statistical Analyses

Data for 17 participants were excluded from the study because the participants withdrew due to illness, or injury (experimental group, *n* = 4; control group *n* = 6) or missed 20% or more of the exercise program (experimental group, *n* = 7). Thus, data for the remaining 73 participants (39 in the experimental group and 34 in the control group) were used in the following analyses. All statistical analyses were performed using SPSS, version 22.0 software (IBM, Inc., Armonk., NY, USA), with a family-wise alpha threshold for all tests set at *p* = 0.05. The normal distribution of the data was confirmed using the Shapiro–Wilk test. Regarding demographic data, independent sample Student's *t*-tests were performed for age, height, weight, and body mass index (BMI) and chi-square tests were used for gender to ensure homogeneity between groups.

For functional fitness data, two-way *(group level: experimental, control)* × *(time level: pre-test, post-test)* repeated measures analyses of variance (ANOVA) were performed for the back scratch, chair sit-and-reach, arm curl, chair stand, 2-min step, and 8-foot up-and-go test results. If significant interactions were identified for the main effects, *post-hoc* tests of the simple effects were performed using paired Student's *t*-tests for within-group analyses or independent Student's *t*-tests for between-group analyses. Partial eta-squared ($\eta_{\text{p}}^{2}$) and Cohen's d (*d*) values were reported for the effect sizes identified in ANOVA and Student's *t*-tests, respectively.

## Results

### Analyses of Descriptive Data

There were no significant differences in baseline gender, age, height, weight, or BMI between the two groups (Table 1).

**Table 1:** Demographic characteristics of participants.

**Variables**	**Total sample**	**Experimental, M (SD)**	**Control, M (SD)**	***p*-value**
	***n* = 73**	***n* = 39**	***n* = 34**	
Male/Female (%)^a^		10/29 (74.4%)	7/27 (79.4%)	0.61
Age (years)	70.36 ± 4.51	69.39 ± 3.60	71.44 ± 5.19	0.06
Height (cm)	158.29 ± 7.77	159.08 ± 8.21	157.38 ± 7.23	0.35
Weight (kg)	58.89 ± 9.64	59.22 ± 11.05	58.51 ± 7.88	0.83
BMI (kg/m^2^)	23.46 ± 2.95	23.35 ± 3.41	23.59 ± 2.34	0.51

*M, mean; SD, standard deviation; BMI, body mass index.*

a *chi-square tests*.

### Effects of Facebook Remote Live-Streaming-Guided Exercise Program on Functional Fitness

For the back scratch test, there were no significant main effects of *group* (*F*_1, 71_ = 1.95, *p* = 0.17), *time* (*F*_1, 71_ = 0.91, *p* = 0.34), or the *group* × *time* interaction (*F*_1, 71_ = 0.47, *p* = 0.50).

For the chair sit-and-reach test, there was a significant main effect of the *group* × *time* interaction (*F*_1, 71_ = 8.80, *p* = 0.004, $\eta_{\text{p}}^{2}$ = 0.11). *Post-hoc* analyses of the simple main effects indicated a significant improvement from pre-test to post-test only in the experimental group (*t*_(38)_ = −2.85, *p* = 0.007, *d* = 0.29). There were no significant effects of *group* (*F*_1, 71_ = 0.29, *p* = 0.59) or *time* (*F*_1, 71_ = 1.04, *p* = 0.31).

An analysis of the arm curl test showed no significant main effects of *group* (*F*_1, 71_ = 0.26*, p* = 0.87), *time* (*F*_1, 71_ = 0.27, *p* = 0.60), or the *group* × *time* interaction (*F*_1, 71_ = 0.64, *p* = 0.43).

For the chair stand test, there were significant main effects of *time* (*F*_1, 71_ = 25.58, *p* < 0.001, $\eta_{\text{p}}^{2}$ = 0.27) and the *group* × *time* interaction (*F*_1, 71_ = 28.09, *p* < 0.001, $\eta_{\text{p}}^{2}$ = 0.28). *Post-hoc* analyses of the simple main effects showed a significant improvement from pre-test to post-test only in the experimental group (*t*_(38)_ = −8.99, *p* < 0.001, *d* = 0.74). In addition, the experimental group performed better post-test than the control group (*t*_[71]_ = −6.35, *p* = 0.005, *d* = 0.69). There was no significant effect of *group* (*F*_1, 71_ = 1.43, *p* = 0.24).

Analysis of the 2-min step test showed that there were significant main effects of *time* (*F*_1, 71_ = 36.25, *p* < 0.001, $\eta_{\text{p}}^{2}$ = 0.34) and the *group* × *time* interaction (*F*_1, 71_ = 14.17, *p* < 0.001, $\eta_{\text{p}}^{2}$ = 0.17). *Post-hoc* analyses of the simple main effects indicated that the experimental group demonstrated a significant improvement from pre-test to post-test (*t*_(38)_ = −8.91, *p* < 0.001, *d* = 0.84). There was no significant effect of *group* (*F*_1, 71_ = 0.32, *p* = 0.58).

For the 8-foot up-and-go test, there were no significant main effects of *group* (*F*_1, 71_ = 3.83, *p* = 0.06), *time* (*F*_1, 71_ = 0.39, *p* = 0.54), or the *group* × *time* interaction (*F*_1, 71_ = 0.02, *p* = 0.97). Table 2 shows a summary of the results of the functional fitness tests.

**Table 2:** Summary of the functional fitness of participants.

**Variables**	**Group**	**Pre-test**	**Post-test**	**Sources**	***F***	***p*-value**	** $\eta_{\text{p}}^{2}$ **
		***M (SD)***	***M (SD)***				
Back scratch (cm)	Experimental	0.15 ± 9.51	−0.38 ± 8.24	Group	1.95	0.17	0.03
	Control	−2.46 ± 10.20	−3.60 ± 11.53	Time	0.91	0.34	0.01
				Group × Time	0.47	0.50	0.01
Chair sit-and-reach (cm)	Experimental	7.01 ± 8.72	9.37 ± 7.71	Group	0.29	0.59	0.00
	Control	9.89 ± 10.57	8.74 ± 9.65	Time	1.04	0.31	0.01
				Group × Time	8.80	0.004^*^	0.11
Arm curl (times)	Experimental	24.28 ± 5.12	26.61 ± 4.68	Group	0.26	0.87	0.00
	Control	25.88 ± 3.57	25.18 ± 3.01	Time	0.27	0.60	0.00
				Group × Time	0.64	0.43	0.01
Chair stand (times)	Experimental	21.03 ± 3.59	23.54 ± 3.32	Group	1.43	0.24	0.02
	Control	21.38 ± 3.84	21.32 ± 3.10	Time	25.58	<0.001^*^	0.27
				Group × Time	28.09	<0.001^*^	0.28
2-min step (times)	Experimental	103.08 ± 11.35	113.28 ± 12.24	Group	0.32	0.58	0.00
	Control	108.44 ± 12.31	110.79 ± 11.14	Time	36.25	<0.001^*^	0.34
				Group × Time	14.17	<0.001^*^	0.17
8-foot up-and-go (sec)	Experimental	5.01 ± 0.66	5.04 ± 0.60	Group	3.83	0.06	0.05
	Control	5.19 ± 0.49	5.24 ± 0.51	Time	0.39	0.54	0.04
				Group × Time	0.02	0.97	0.00

*M, mean; SD, standard deviation.*

* *p < 0.05*.

### Additional Analysis

We performed an additional analysis including the participants in the experimental group who missed 20% or more of the exercise program. Including these participants in the analysis did not alter the results, indicating that only the experimental group demonstrated a significant improvement in the chair sit-and-reach (*t*_[45]_ = −2.48, *p* = 0.017, *d* = 0.29), chair stand (*t*_[45]_ = −0.71, *p* < 0.001, *d* = 0.87), and 2-min step tests (*t*_[45]_ = −0.86, *p* < 0.001*, d* = 0.88) from pre-test to post-test.

## Discussion

The aim of this study was to determine the effect of an 8-week Facebook remote live-streaming-guided exercise program on the functional fitness of community-dwelling older adults. Our findings suggested that this program is a feasible means of improving lower limb flexibility and muscle strength and cardiorespiratory fitness in community-dwelling older adults.

The results of the current study partly aligned with those of previous studies, showing that home-based tele-exercise has positive effects on the functional fitness of older adults (18, 19). A previous study demonstrated that tele-based exercise increases lower limb flexibility and muscle mass (18). In another study, elderly women with a risk of falling exhibited improved lower limb muscle performance after 12 weeks of a web-based exercise program delivered via a telepresence platform (19). Any discrepancies in the results of these studies may be due to small sample sizes or differences in participants' characteristics (e.g., participants with or without a high risk of falling). In the current study, we conducted an 8-week live-streaming exercise program via Facebook and found that such an intervention concurrently increased lower limb flexibility, muscle strength, and cardiorespiratory fitness in older adults. Using the live-streaming method, trainers provided real-time instructions and feedback during the exercise intervention, which may have increased the effectiveness of the exercise program at improving the functional fitness of older adults. Previous studies have demonstrated broad improvements in functional fitness in older adults after traditional exercise interventions (30–32). In response to the COVID-19 pandemic, many countries have implemented policies for social distancing and varying degrees of quarantine or isolation at home (13). Such strategies have had unintended negative effects on the functional fitness of older adults (33, 34) due to decreased physical activity and increased sedentary behavior (14, 15). Therefore, based on the results of this study, we propose that live-streaming home-based exercise programs may be an effective alternative method for increasing the functional fitness of community-dwelling older adults during the pandemic.

We found no significant positive effects on balance, agility, or upper limb flexibility or muscle mass following 8 weeks of the Facebook live-streaming exercise program. A previous study found that older adults had a higher gait speed and improved balance after 12 weeks of an iPad-based training program (35). A 15-week tai chi video-based exercise program was shown to improve balance in elderly individuals (36). Furthermore, older adults in another study demonstrated improved upper limb flexibility, muscle strength, balance and agility after 12, but not 8, weeks of Thai yoga exercise (37). Such findings suggest that relatively long periods of exercise intervention may be necessary to improve the balance and agility of older adults. Many of the movements in the current exercise program, such as aerobic dance, calf raises, and standing on one foot, involved the lower limbs. That is, the majority of the exercise program focused on the lower limbs rather than the upper limbs. To increase the effectiveness of exercise programs at improving the functional fitness of older adults, further studies should consider the duration of the intervention when designing the program.

There were several limitations of this study that must be acknowledged. First, this study used a non-randomized controlled design. Nevertheless, the demographic and functional fitness data were homogenous between the two groups. Second, the intensity of the exercise intervention was unknown. In the current study, participants had increased cardiorespiratory fitness following completion of the exercise program. Given that the American College of Sports Medicine guidelines for older adults suggest that at least moderate-intensity exercise is necessary to increase cardiorespiratory fitness and muscle strength (38), the exercise intensity of the intervention in this study may have been at least moderate. However, future studies should clearly set the exercise intensity using objective (heart rate or a one-repetition maximum test) or subjective (rating of perceived exertion) measurements.

## Conclusions

The results of the current study indicate that a home-based, remote live-streaming-guided exercise program delivered via the Facebook platform may be a practical approach to improve the functional fitness of community-dwelling older adults.

## Data Availability Statement

The raw data supporting the conclusions of this article will be made available by the authors, without undue reservation.

## Ethics Statement

The studies involving human participants were reviewed and approved by the Research Ethics Committee of the National Taiwan Normal University. The patients/participants provided their written informed consent to participate in this study. Written informed consent was obtained from the individual(s) for the publication of any potentially identifiable images or data included in this article.

## Author Contributions

S-HC, L-TW, and T-YC: study concept and design and drafting of the manuscript. L-TW, M-CH, and Y-WW: acquisition of data. L-TW, T-YC, and T-MH: analysis and interpretation of data. T-YC, M-CH, and T-MH: critical revision of the manuscript for important intellectual content. All authors contributed to the article and approved the submitted version.

## Funding

This study was financially supported by the Ministry of Science and Technology of Taiwan (MOST 108-2410-H-003-117).

## Conflict of Interest

The authors declare that the research was conducted in the absence of any commercial or financial relationships that could be construed as a potential conflict of interest.

## Publisher's Note

All claims expressed in this article are solely those of the authors and do not necessarily represent those of their affiliated organizations, or those of the publisher, the editors and the reviewers. Any product that may be evaluated in this article, or claim that may be made by its manufacturer, is not guaranteed or endorsed by the publisher.

## References

[1] United Nations. World Population Prospects: The 2019 Revision. New York, NY: United Nations (2020). Retrieved from: https://population.un.org/wpp/

[2] Chung PK Zhao Y Liu JD Quach B. A canonical correlation analysis on the relationship between functional fitness and health-related quality of life in older adults. Arch Gerontol Geriatr Gerontol Int. (2017) 68:44–8. 10.1016/j.archger.2016.08.007 27620501

[3] Brown DS Thompson WW Zack MM Arnold SE Barile JP. Associations between health-related quality of life and mortality in older adults. Prevent Sc. (2015) 16:21–30. 10.1007/s11121-013-0437-z 24189743 PMC4593240

[4] Rikli RE Jones CJ. Senior Fitness Test Manual. 2nd edn. Champaign, IL: Human Kinetics (2013).

[5] Zhao Y Chung P. Differences in functional fitness among older adults with and without risk of falling. Asian Nurs Res. (2016) 10:51–5. 10.1016/j.anr.2015.10.007 27021835

[6] Kim Hj Park I joo Lee H Lee O. The reliability and validity of gait speed with different walking pace and distances against general health, physical function, and chronic disease in aged adults. J Exerc Nutr Biochem. (2016) 20:46. 10.20463/jenb.2016.09.20.3.7 27757387 PMC5067420

[7] Yang M Guo Y Gong J Deng M Yang N Yan Y. Relationships between functional fitness and cognitive impairment in Chinese community-dwelling older adults: a cross-sectional study. BMJ Open. (2018) 8:e020695. 10.1136/bmjopen-2017-020695 29780027 PMC5961618

[8] Lee J Lau S Meijer E Hu P. Living longer, with or without disability? a global and longitudinal perspective. J Gerontol Series A. (2020) 75:162–7. 10.1093/gerona/glz007 30629214 PMC6909890

[9] Yang YP Lin HC Chen KM. Functional fitness in older adults: a systematic review and meta-analysis. Top Geriatr Rehabil. (2019) 35:238–47. 10.1097/TGR.000000000000024130661187

[10] Physical Activity Guidelines for Americans. 2nd ed. U.S. Department of Health and Human Services (2018). Available online at: https://health.gov/sites/default/files/2019-09/Physical_Activity_Guidelines_2nd_edition.pdf

[11] Boulton ER Horne M Todd C. Multiple influences on participating in physical activity in older age: developing a social ecological approach. Health Expect. (2018) 21:239–48. 10.1111/hex.12608 28768065 PMC5750764

[12] Santos DA Silva AM Baptista F Santos R Vale S Mota J. Sedentary behavior and physical activity are independently related to functional fitness in older adults. Exp Gerontol. (2012) 47:908–12. 10.1016/j.exger.2012.07.011 22884978

[13] World Health Organization. COVID-19 Strategy Update. (2020). Available online at: https://www.who.int/publications/m/item/covid-19-strategy-update (accessed April 14, 2020).

[14] López-Sánchez GF López-Bueno R Gil-Salmerón A Zauder R Skalska M Jastrzebska J. Comparison of physical activity levels in Spanish adults with chronic conditions before and during COVID-19 quarantine. Eur J Public Health. (2021) 31:161–6. 10.1093/eurpub/ckaa159 32761181 PMC7454536

[15] Schuch F Bulzing R Meyer J López-Sánchez G Grabovac I Willeit P. Moderate to vigorous physical activity and sedentary behavior changes in self-isolating adults during the COVID-19 pandemic in Brazil: a cross-sectional survey exploring correlates. Sport Sci Health. (2021). 10.1007/s11332-021-00788-x. [Epub ahead of print]. 34108999 PMC8179086

[16] Gilmour J Machin T Brownlow C Jeffries C. Facebook-based social support and health: a systematic review. Psychol Popular Media. (2020) 9:328. 10.1037/ppm0000246

[17] Napoleon. Facebook Users in Taiwan. (2020). Available online at: https://napoleoncat.com/stats/facebook-users-in-taiwan/2019/11/ (accessed November, 2019).

[18] Hong J Kim J Kim SW Kong HJ. Effects of home-based tele-exercise on sarcopenia among community-dwelling elderly adults: body composition and functional fitness. Exp Gerontol. (2017) 87:33–9. 10.1016/j.exger.2016.11.002 27838369

[19] Hong J Kong HJ Yoon HJ. Web-based telepresence exercise program for community-dwelling elderly women with a high risk of falling: randomized controlled trial. JMIR mHealth uHealth. (2018) 6:e132. 10.2196/mhealth.9563 29807877 PMC5996181

[20] Mirmoezzi M Amini M Khaledan A Khorshidi D. Effect of 8-week of selected aerobic exercise on static and dynamic balance in healthy elderly inactive men. Iran J Ageing. (2016) 11:202–9. 10.21859/sija-1101202

[21] Baker BS Miller K Weitzel KJ Duren DL Gammon R Mills-Gray S. Resistance training reduces age-and geography-related physical function discrepancies in older adults. Gerontol Geriatr Med. (2021) 7:2333721421992251. 10.1177/2333721421992251 33614831 PMC7868454

[22] Park J McCaffrey R Newman D Liehr P Ouslander JG. A pilot randomized controlled trial of the effects of chair yoga on pain and physical function among community-dwelling older adults with lower extremity osteoarthritis. J Am Geriatr Soc. (2017) 65:592–7. 10.1111/jgs.14717 28008603 PMC5357158

[23] Thompson WR Gordon NF Pescatello LS. ACSM's Guidelines for Exercise Testing and Prescription. Philadelphia: Wolters Kluwer Health/Lippincott Williams & Wilkins (2010).

[24] Toraman NF Erman A Agyar E. Effects of multicomponent training on functional fitness in older adults. J Aging Phys Act. (2004) 12:538–53. 10.1123/japa.12.4.538 15851825

[25] De Resende-Neto A do Nascimento M da Silva D Netto R de Santana J Silva M. Effects of multicomponent training on functional fitness and quality of life in older women: a randomized controlled trial. Int J Sport Exerc Med. (2019) 5. 10.23937/2469-5718/1510126

[26] Martinez-Navarro I Cordellat A Roldán A Sanchis G Blasco-Lafarga C. 120 min/week of neuromotor multicomponent training are enough to improve executive function and functional fitness in older women. Exp Gerontol. (2021) 145:111199. 10.1016/j.exger.2020.111199 33310154

[27] Chodzko-Zajko WJ Proctor DN Singh MAF Salem GJ Skinner JS. Exercise and physical activity for older adults. Med Sci Sports Exercise Special Comun. (2009) 41:1510–30. 10.1249/MSS.0b013e3181a0c95c 19516148

[28] American College of Sports Medicine. In: ONG HS, editor. Exercise and Physical Activity for Older Adults. (2009). Retrieved from: http://beta.acsm.org/docs/default-source/default-document-library/acsm-positions-and-policy/translated-position-stands/chinese/ct_older_adults.pdf?sfvrsn=e0a2e78_2

[29] Rikli RE Jones CJ. Development and validation of a functional fitness test for community-residing older adults. J Aging Phys Act. (1999) 7:129–61. 10.1123/japa.7.2.129

[30] Hwang CL Lim J Yoo J-K Kim HK Hwang MH Handberg EM. Effect of all-extremity high-intensity interval training vs. moderate-intensity continuous training on aerobic fitness in middle-aged and older adults with type 2 diabetes: a randomized controlled trial. Exp Gerontol. (2019) 116:46–53. 10.1016/j.exger.2018.12.013 30576716 PMC6404965

[31] Martins R Pindus D Cumming S Teixeira A Veríssimo M. Effects of strength and aerobic-based training on functional fitness, moodand the relationship between fatness and mood in older adults. J Sports Med Phys Fitness. (2011) 51:489–96. 21904289

[32] DiBrezzo R Shadden BB Raybon BH Powers M. Exercise intervention designed to improve strength and dynamic balance among community-dwelling older adults. J Aging Phys Act Instr Older Adults. (2005) 13:198–209. 10.1123/japa.13.2.198 15995265

[33] Cheval B Sieber S Maltagliati S Millet GP Formánek T Chalabaev A. Muscle strength is associated with COVID-19 hospitalization in adults 50 years of age and older. MedRxiv [Preprint]. (2021) 1–21. 10.1101/2021.02.02.21250909 34363345 PMC8426913

[34] Kirwan R McCullough D Butler T de Heredia FP Davies IG Stewart C. Sarcopenia during COVID-19 lockdown restrictions: long-term health effects of short-term muscle loss. GeroScience. (2020) 42:1547–78. 10.1007/s11357-020-00272-3 33001410 PMC7528158

[35] Silveira P Daniel F Casati F. Tablet-based strength-balance training to motivate and improve adherence to exercise in independently living older people: part 2 of a phase II preclinical exploratory trial. J Med Inter Res. (2014) 16:e159. 10.2196/jmir.3055 24966165 PMC4090377

[36] Wu GE Keyes LM. Group tele-exercise for improving balance in elders. Telemed J E Health. (2006) 12:561–70. 10.1089/tmj.2006.12.561 17042710

[37] Noradechanunt C Worsley A Groeller H. Thai Yoga improves physical function and well-being in older adults: a randomised controlled trial. J Sci Med Sport. (2017) 20:494–501. 10.1016/j.jsams.2016.10.007 27866841

[38] American College of Sports Medicine. ACSM's Exercise Testing and Prescription. 10th ed. New York, NY: Lippincott Williams & Wilkins (2017).

